# Effects of cancer-testis antigen, TFDP3, on cell cycle regulation and its mechanism in L-02 and HepG2 cell lines *in vitro*

**DOI:** 10.1371/journal.pone.0182781

**Published:** 2017-08-10

**Authors:** Yunshen Jiao, Lingyu Ding, Ming Chu, Tieshan Wang, Jiarui Kang, Xiaofan Zhao, Huanhuan Li, Xi Chen, Zirui Gao, Likai Gao, Yuedan Wang

**Affiliations:** 1 Department of Immunology, School of Basic Medical Science, Peking University, Beijing, China; 2 Key Laboratory of Medical Immunology, Ministry of Health, Beijing, China; 3 Beijing Research Institute of Chinese Medicine, Beijing University of Chinese Medicine, Beijing, China; 4 Department of Pathology, the First Affiliated Hospital of General Hospital of Chinese People’s Liberation Army, Beijing, China; University of South Alabama Mitchell Cancer Institute, UNITED STATES

## Abstract

TFDP3, also be known as HCA661, was one of the cancer-testis antigens, which only expressed in human tissues. The recent researches about TFDP3 mostly focused on its ability to control the drug resistance and apoptosis of tumor cells. However, the role of TFDP3 in the progress of the cell cycle is rarely involved. In this study, we examined the expression of TFDP3 in human liver tissues firstly. After that, we detect the expression of TFDP3 at the RNA level and protein level in L-02 cell line and HepG2 cell line, and the location of TFDP3 was defined by immunofluorescence technique. Furthermore, we synchronized the cells to G1 phase, S phase and G2 phase, and arrested cell mitosis. The localization of TFDP3 and co-localization with E2F1 molecules in different phases of hepatocyte lines. Finally, TFDP3 gene knockout was performed on L-02 and HepG2 cell lines, and detected the new cell cycles by flow cytometry. The result showed that the expression of TFDP3 molecule is negative in normal liver tissue, but positive in immortalized human hepatocyte cell line, and the expression level is lower than in hepatocellular carcinoma cell line. The expression level of TFDP3 was in the dynamic change of L-02 and HepG2 cell lines, and was related to the phase transition. TFDP3 can bind to E2F1 molecule to form E2F/TFDP3 complex; and the localizations of TFDP3 and E2F1 molecules and the co-localization were different in different phases of cell cycle in the nucleus and cytoplasm, which indicated that the E2F/TFDP3 complex involved in the process of regulating the cell cycle. By knocking down the TFDP3 expression level in L-02 and HepG2 cell lines, the cell cycle would be arrested in S phase, which confirmed that TFDP3 can be a potential target for tumor therapy.

## Introduction

In the 1980s, the activation factor of the adenovirus E2 promoter (E2F) was found, which could interact with various cell cycle-dependent proteins, such as retinoblastoma (Rb) proteins. E2Fs play an important role on regulating cell cycle from G1 phase into the S phase[1], so as the process of cell proliferation, differentiation, apoptosis and autophagy. In 1993, Girling et al.[2] isolated a highly homologous polypeptide molecule from the E2F complex and named it as transcription factor dimerization partner (TFDP/DP) molecule[3]. Although DP protein molecules are crucial for E2F/TFDP complex, the study and understanding of the structure and function of DP protein family members are far less clear than E2F family members.

Studies have shown that E2Fs play an important role in cell cycle and tumorigenesis by regulating the expression of genes required for cell differentiation, proliferation, apoptosis, DNA repair and DNA replication[4]. The transcription factor of the E2F family is closely related to the cell cycle progression and apoptosis by coordinating the expression of a large number of genes involved in the regulation of G1 to S phase transformation, as well as other genes involved in apoptosis[5]. The E2F/TFDP complex recognizes promoter regions of approximately 1240 genes, thereby acting to activate or inhibit gene transcription[6]. It is generally believed that the consensus sequence of these promoter regions is TTT (C/G) GCGC (C/G), which is the region identified by the E2F/TFDP complex[7].

At present, it is generally believed that E2F family includes eight members. E2F1 is the earliest discovery and the most representative E2F molecule, which can form a complex with TFDP1 to regulate the transcription and expression of downstream target gene[8], causing quiescent cells to enter the S phase or to promote cell into the apoptosis process. Therefore, E2F1 is also the only molecule which can both promote cell proliferation and apoptosis in E2F family[9].

There have been found three TFDP family members: TFDP1, TFDP2 and TFDP3. Their sequences have high homology[10]. Being different with the other TFDP family members, TFDP3 is a human-specific TFDP family of protein molecules[11], and expressed only in malignant tumor cells such as melanoma, liver cancer, breast cancer and T lymphocytic leukemia, also in normal testis tissues. The domain of testicular antigens is a good target molecule for genetically engineered T cells in the treatment of tumors[12], for TFDP3 is a cancer-testis antigen.

Since the TFDP3 molecule was found, it was attractive because of its close relationship with the organization and function of E2F/TFDP complex. On one hand, as a cancer-testis antigen, TFDP3 has good immunogenicity, and is expressed only in tumor cells and testicular spermatogenic cells (which not express HLA molecules), is a good specific CTL target[13]. The vaccine made by specific antigen peptide sequences could excite the body-specific CTL-mediated anti-tumor immunity, but could not induce autoimmunity. On the other hand, as a cancer-testis antigen molecule with clear functions, TFDP3 is related to the occurrence and development of cancers and germ cell development. There is a series of papers on the function of TFDP3 protein[14] clarified that TFDP3 could compete with TFDP1 and bind to E2F1 to form E2F1/TFDP3 complex. The complex cannot be conjugated with TTT(C/G)GCGC(C/G), which could be recognized by the traditional E2F/TFDP complex, so that E2F1 lost the function of initiating apoptosis-related gene expression, therefore blocking the E2F1-induced apoptosis process[15], so as to promote cell survival.

It has been clarified that E2F1 / TFDP complex formation is one of the key aspects of cell cycle regulation. When cells entering the S phase from the G1 phase, the cell cycle regulatory proteins (such as CDK1) could phosphorylate the Rb protein bounding to the E2F1/TFDP complex to form a phosphorylated Rb protein (pRb). After that, pRb falls off from the complex, therefore to induce the complex into the nucleus. Moreover, it could combine with target gene promoters with TTT(C/G) GCGC (C/G) to initiate the transcription and expression of them, and promote the cell into S phase from G1 phase. On the contrary, TFDP3 can compete with TFDP1 to combine with E2F1, which would interfere with the regulatory activity of E2F1 in the cell cycle. The overexpression of TFDP3 may cause the active inhibition of E2F1 and resulting in cell cycle block.

Whether TFDP3 molecular could participate in the process of cell cycle or not is an attractive project. In this study, we explored the expression of TFDP3 in normal liver and liver cancer tissues, and in the immortalized hepatocyte line L-02 and hepatocellular carcinoma cell line HepG2, to discuss the role and mechanism of TFDP3 in cell cycle.

## Materials and methods

### Immunohistochemistry

Tissue microarrays were dewaxed by soaking twice in xylene for 10 min. Next, the slide was sequentially immersed in 100%, 95%, 85% and 70% ethanol for 5 min each; immersed in distilled water for 5 min; and then washed twice with PBS for 5 min. The microarray was incubated with 3% H_2_O_2_ at room temperature for 10 min and then washed twice with PBS for 5 min. Antigen retrieval was carried out twice in 0.01 M sodium citrate buffer, and the slide was then cooled at room temperature for approximately 15–30 min before it was washed twice with PBS for 5 min. The microarray was incubated in blocking solution for 20–30 min at room temperature followed by a primary antibody (1:50) at room temperature for 1–2 hours. After the microarray was washed twice with PBS, it was incubated in secondary antibody at room temperature for 15–30 minutes and then washed twice again. The DAB reagent was added at room temperature for 3–5 minutes, and the microarray was washed with distilled water. Finally, hematoxylin was added, and the microarray was washed with distilled water. The microarray was prepared and observed under a microscope[16].

### Cell culture and transfection

Human liver carcinoma cell line HepG2 was purchased from ATCC. The normal human hepatocyte cell line L-02 was purchased from the Type Culture Collection of the Chinese Academy of Sciences, Shanghai, China. HepG2 and L-02 cell lines were maintained in Dulbecco’s modified Eagle’s medium(DMEM) with 10% (v/v) fetal bovine serum (FBS), 0.1 mM nonessential amino acids, 100 U/ml penicillin, and 100 μg/ml streptomycin at 37°C in a 5% CO_2_ incubator[17]. Lipofectamine 3000 reagent (Invitrogen) was used for the delivery of siRNA into cells. The TFDP3-siRNA sequences had been patented, therefore not described here.

### Real-time PCR

TransStart Tip Green qPCR SupreMix was purchased from TransGen Biotech, and all of the procedures were carried out according to the manufacturer’s protocol. For TFDP3, the forward primer was 5’-ATGGACGAGAACCAGACCAG-3’ and the reverse primer was 5’-CCCAGACCTTCATGGAAAGA-3’. For GAPDH, the forward primer was 5’-AATGACCCCTTCATTGAC-3’ and the reverse primer was 5’-TCCACGACGTACTCAGCGC-3’. The cycling parameters were 94°C for 15 s, 60°C for 30 s and 72°C for 60 s; 40 cycles were carried out[2].

### Western blotting

Cells were harvested and lysed for protein extraction followed by the determination of protein concentration. The supernatant was used for Western blotting. Proteins were separated by SDS-PAGE and transferred onto polyvinylidene fluoride (PVDF) membranes. The membranes were incubated with primary antibodies, including anti-TFDP3 and anti-GAPDH (Santa Cruz Biotechnology, CA, USA), followed by incubation with secondary antibodies conjugated to HRP. Signal development was performed with an ECL kit (QIAGEN). Each experiment was performed three times[18].

### Immunofluorescence staining

Cells were washed three times with PBS and were then fixed with 4% pre-cooled paraformaldehyde for 15 min. The cells were washed with PBS and then permeabilized with 0.1% triton-X100 (diluted in PBS) for 20 min at room temperature. The cells were washed with PBS (containing 0.1% triton) as described above and were then incubated with 5% bovine serum albumin (BSA) for 30 min at room temperature. The coverslips were dried with absorbent paper and incubated with primary antibody overnight at 4°C. The cells were washed with PBS (containing 0.1% triton) as stated above, incubated with a fluorescence-conjugated secondary antibody at 37°C for 1 h in the dark and then washed. FITC-labeled goat anti-rabbit secondary antibody (EarthOx) was used to bind E2F1 rabbit polyclonal antibody (Abcam), and Cy3-labeled goat anti-mouse secondary antibody (EarthOx) was used to bind TFDP3 monoclonal antibody (Santa Cruz). The cells were incubated with DAPI for 90 seconds, washed with PBS and then prepared for observation under a fluorescence microscope (Olympus).

### Cell cycle synchronization

Prepare the DMEM medium with thymidine (Sigma) 100 mmol/L, and dilute it in DMEM complete medium(with 10%FBS) after incubation so that the final concentration of thymidine was 2 mmol/L. After culturing HepG2 and L-02 cells in the 6-wells culture plate, remove the old medium and wash the cells with PBS three times, and culture the cells with DMEM complete medium containing 2mmol/L thymidine for 16 hours. Afterward, the old medium should be removed and the cells were washed with PBS three times, and the cells cultured in fresh DMEM complete medium for 10 h continuingly. Then the old medium should be removed and the cells were washed with PBS three times, and re-adding DMEM complete medium with 2mmol/L TdR in the plate, and continuing to culture the cells for 16 hours. The cells were blocked in G1 phase at the moment. Removing the old medium and culturing the cells with fresh DMEM complete medium for 4 h and 8 h to collect the cells in S phase and G2 phase. Trypsin can be used to harvest cells. Cells cultured in DMEM complete medium containing 40 ng/mL nocodazole for 16 hours. The cells in M phase could shed off from the plate by gently shaking the plate.

### Cell cycle detection

Samples were prepared for detection in a flow cytometer (BD, Canton II), and all of the procedures were carried out according to the manufacturer’s protocol. The samples were carefully shaken before loading to avoid cell clusters. The speed for loading the cells was slow, and 10000 cells were collected from each tube. The samples were loaded into the flow cytometer as soon as possible after the preparation procedure. Samples were kept in the dark to avoid fluorescence quenching.

### Statistical analysis

All data are presented as mean±standard deviation. Student's t-test was used for two group's comparison. In all cases, P < 0.05 was considered with statistical significant. *p<0.05, **p<0.01 and ***p<0.001 Levene’s test was used to examine the quality of variances. [19]

## Results and discussion

### The expression of TFDP3 in the normal liver tissue was negative

**Fig 1:**
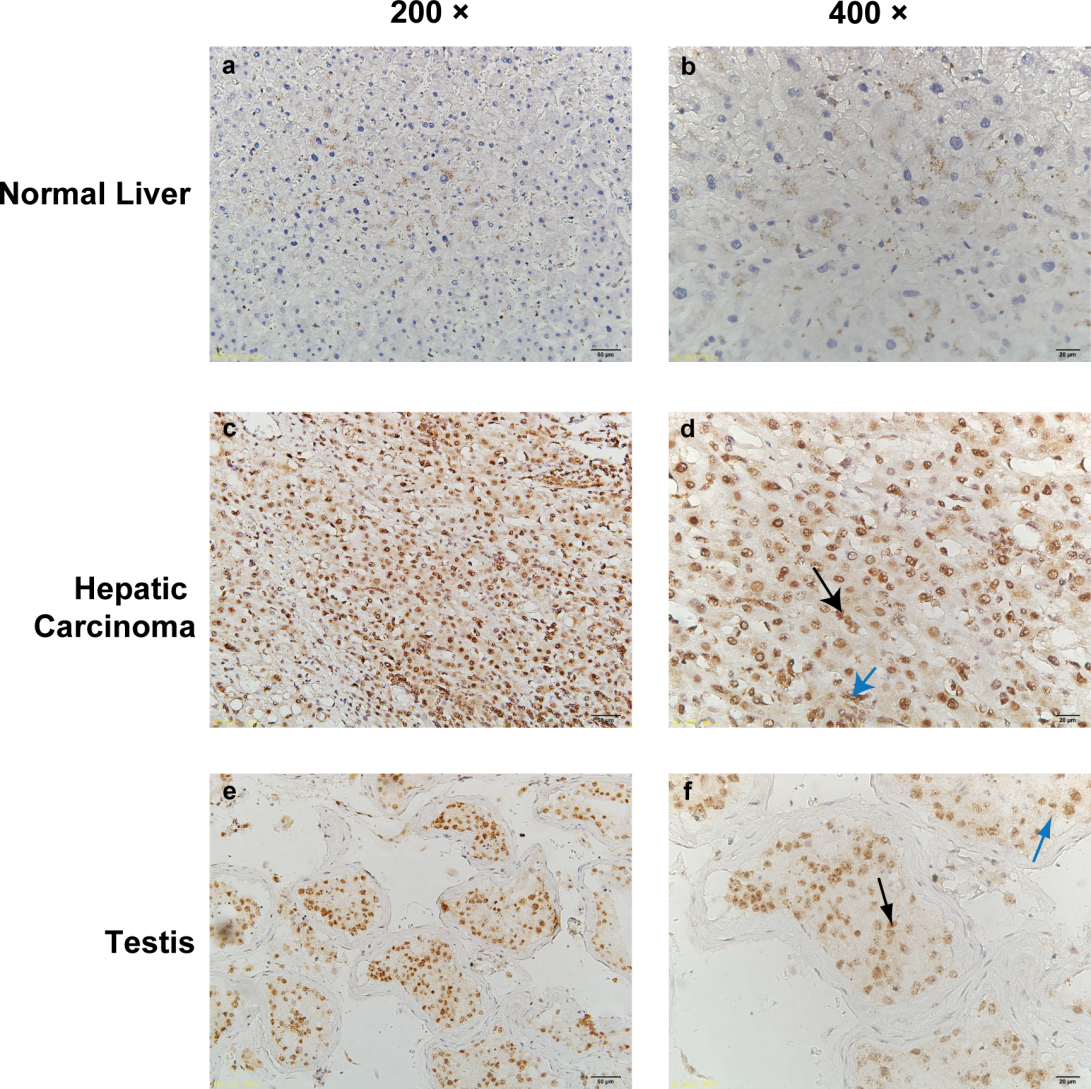
TFDP3 in human normal liver and hepatocellular carcinoma tissue expression. The figure shows the results of immunohistochemical staining of human normal liver (a,b), human hepatocellular carcinoma (c,d) and human testis tissue sections (e,f). Tissue sections were stained with anti-TFDP3 antibody and subjected to hematoxylin staining (a,c,e ×200 and b,d,f ×400). The expression of TFDP3 in human normal liver tissue was negative, while in human hepatocellular carcinoma tissue and testicular tissue TFDP3 molecule was stained in nucleus (as indicated by black arrow in the figure) into brown (high expression), and cytoplasm (indicated by blue arrow) into light brown (low expression).

Immunohistochemistry was used to detect the expression of TFDP3 in human hepatocellular carcinoma and normal liver tissue, and testis tissue was used as positive control. In each sample, 3 to 5 visual field records were randomly selected and the positive rate was calculated. The results showed that the expression of TFDP3 was negative in human normal liver tissue, while the expression of TFDP3 was positive in hepatocellular carcinoma and testis tissues (Fig 1).

### Expression of cancer testis antigen TFDP3 in immortalized human hepatocyte line L-02 and hepatocellular carcinoma cell line HepG2

**Fig 2:**
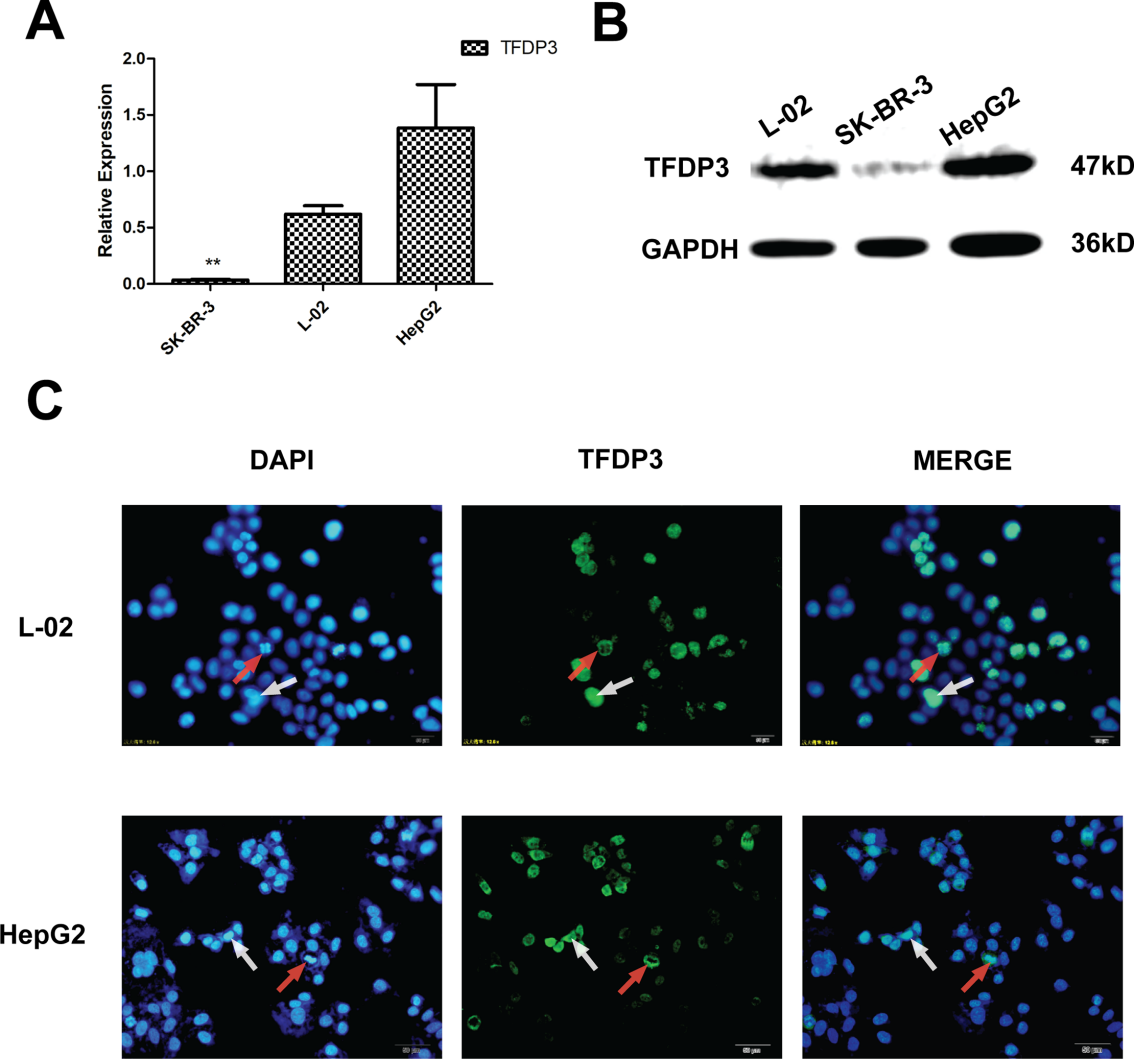
Expression of TFDP3 on L-02 cells and HepG2 cells. (A) The expression of TFDP3 in SK-BR-3, L-02 and HepG2 cell lines in mRNA level was detected by quantitative RT-PCR. TFDP3 and GAPDH primer sequences were shown before. L-02 and HepG2 cell lines expressed TFDP3 at the RNA level, and the expression level of TFDP3 in L-02 cell line was lower than in HepG2 cell line. The expression of TFDP3 in SK-BR-3 cell line was negative. (B) The expression of TFDP3 in SK-BR-3 cells, L-02 cells and HepG2 cells detected by Western Blot, and the expression level of TFDP3 in L-02 cell line was lower than in HepG2 cell line. (C) Immunofluorescence staining was used to detect the localization and expression of TFDP3 in L-02 cells and HepG2 cells. TFDP3 molecules could locate only in cytoplasm (as in the cells in mitotic indicated by red arrow), or also locate in the nucleus (as in indicated by white arrow).

SK-BR-3 cell line were used as negative control in this study. The TFDP3 expression in HepG2 and L-02 cell lines were detected at mRNA level. The results showed that TFDP3 was expressed both in L-02 and HepG2 cell lines at mRNA level (Fig 2A) and at protein level (Fig 2B), and the expression level of TFDP3 was higher in HepG2 cell line (Figure A in S1 Fig). The immunofluorescence staining results showed that the expression of TFDP3 was related to the cell cycle (Fig 2C). It can be seen that in L-02 and HepG2 cell lines, the expression level and position of TFDP3 is different.

### The expression of TFDP3 is characteristics in different cell cycle phases

**Fig 3:**
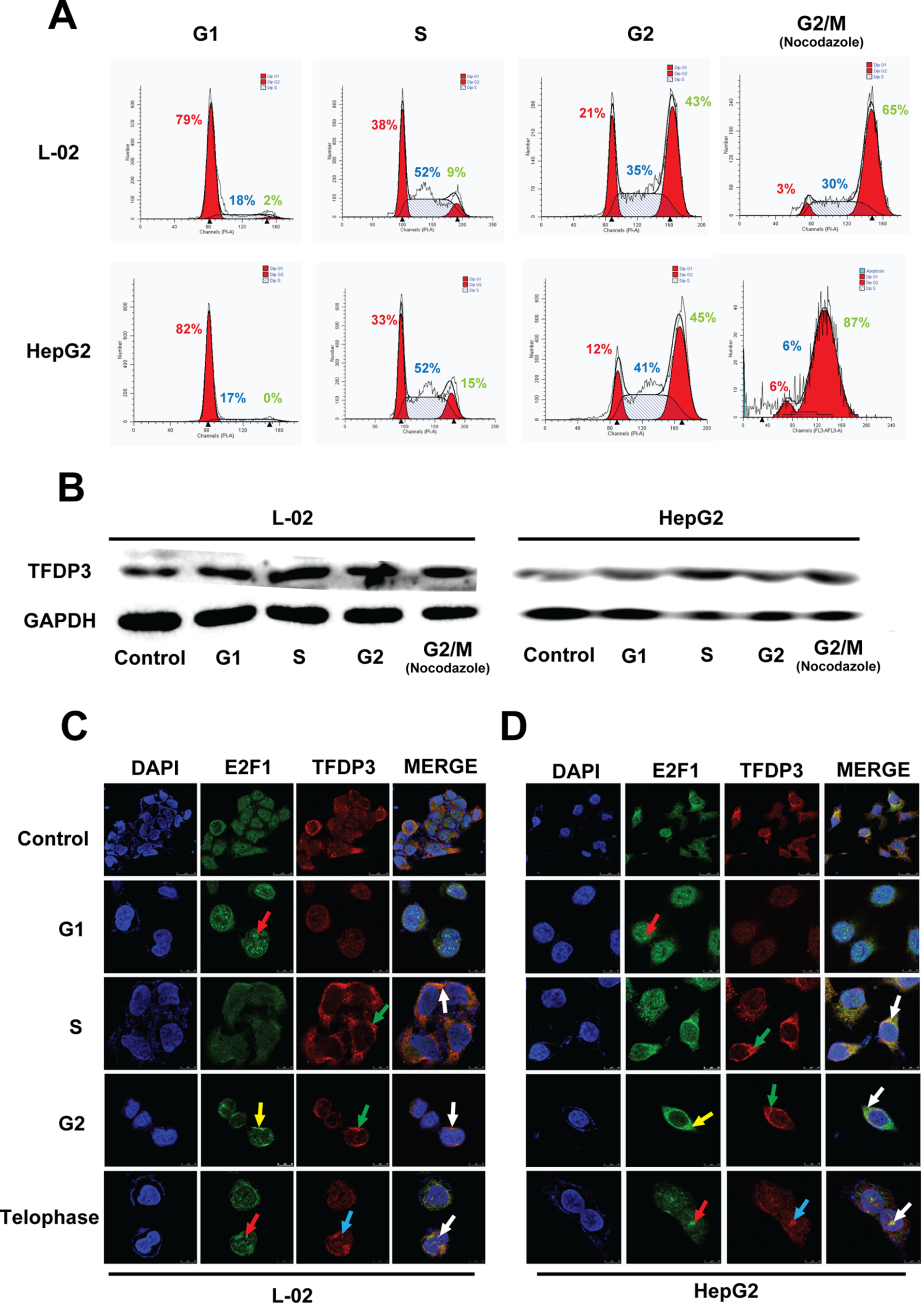
Expression of TFDP3 in L-02 and HepG2 cell lines in different phases of cell cycle. (A) L-02 and HepG2 cell lines were synchronized to G1, S, G2 and M phases through TdR method and nocodazole, and synchronization effect was measured by flow cytometry. The proportion of each phase was shown in the figure (Red number = G1 phase, Blue number = S phase, Green number = G2 phase). This experiment was repeated 5 times to count the proportion of cells in each cell after synchronized. It can be seen that the proportion of cells that are synchronized is about 50% or so. (B) The expression of TFDP3 protein in each phase of cell cycle in L-02 and HepG2 cell lines was detected by Western Blot. The result showed that in L-02 and HepG2 cell lines, TFDP3 was expressed in every phase of cell cycle, and the expression level is various in different phases. Moreover, the expression level in S phase was higher than the other phases (p_L-02_ = 0.003<0.01; p_HepG2_ = 0.007<0.01; n = 5). The results of immunofluorescence staining showed the localization of TFDP3 and E2F1 in different phases of cell cycle in L-02 cell line (C) and HepG2 cell line (D), which were examined by laser confocal scanning microscopy. In the figures, the images from the top to the bottom in turn showed the distribution of E2F1 and TFDP3 in normal untreated cells (8000×), G1 phase, S phase, G2 phase and late stage cells (20000×); from left to right in turn showed nucleus staining (DAPI), E2F1 (FITC tag), TFDP3 (Cy3 tag) and merge images. The red arrows indicate that the location of the E2F1 expressed in nucleus in telophase and G1 phase. The yellow arrows indicate that the E2F1 mainly expressed in cytoplasm in the G2 phase. The blue arrows indicate the expression of TFDP3 in nucleus in telophase. The green arrows indicate that TFDP3 mainly expressed in cytoplasm in S and G2 phase. The co-localization of TFDP3 and E2F1 was indicated by the white arrows.

Thymidine double Repression (TdR) method is known as an effectively way to synchronize cell phase and suitable for most cell culture system; also, the toxicity of TdR method to cells in S phase is relatively small. Therefore we chose to use TdR method to induce cell synchronization to G1 / S / G2 phase. Nocodazole is widely used to block the cells in mitosis and. Flow cytometry was used to detect the synchronization effect (Fig 3A). The results showed that the cell synchronization effects were acceptable in each cycle. The proportion of cells after synchronization changed significantly (Figure B in S1 Fig). Western Blot was used to investigate the expression of TFDP3 in different phases in L-02 and HepG2 cell lines (Fig 3B). The results showed that TFDP3 expressed higher in S phase in both L-02 and HepG2 cell lines (Figure C in S1 Fig, p <0.01). Moreover, we detected the localization of TFDP3 and E2F1 by laser confocal microscopy in every phases of cell cycle in L-02 cell line (Fig 3C) and HepG2 cell line (Fig 3D). Immunofluorescence staining results showed that the localization and the expression level of TFDP3 and E2F1 were not consistent during the cell cycle. E2F1 expressed in the nucleus (as indicated by the red arrow) in G1 phase and in the end of mitosis, expressed diffusively in the cells in S phase, and mainly expressed in the cytoplasm (as indicated by the yellow arrow) in G2 phase. However, TFDP3 expressed in the nucleus only in the end of mitosis (as indicated by the blue arrow), expressed diffusively in the cells in G1 phase, and expressed in the cytoplasm (as indicated by the green arrow) in G2 phase. The expression of TFDP3 and E2F1 was clearly co-localized (as indicated by the white arrow), either in cytoplasm (S and G2 phase) or nucleus (the end of mitosis).

### TFDP3 is involved in the regulation of cell cycle

**Fig 4:**
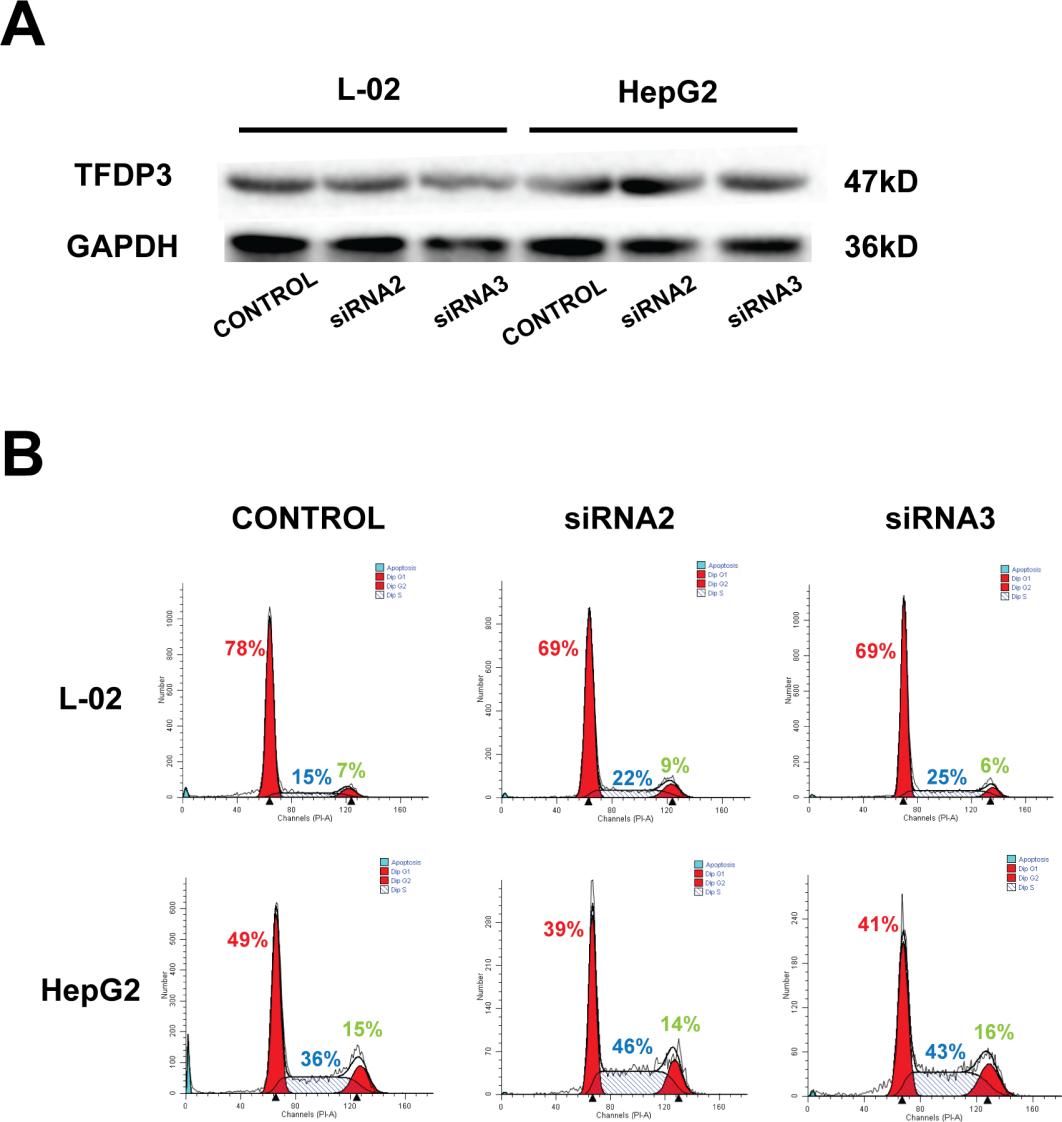
Cell cycle changes of L-02 cell line and HepG2 cell line after TFDP3 knockdown. (A) The expression of TFDP3 in L-02 and HepG2 cell lines transfected with TFDP3-siRNA2 and TFDP3-siRNA3 was detected by Western Blot. (B) Flow cytometry was used to detect cell cycle changes in L-02 and HepG2 cell lines after TFDP3 knockdown (Red number = G1 phase, Blue number = S phase, Green number = G2 phase). It is clearly that the proportion of cells in G1 phase declined, and inclined in S phase.

In this study, siRNA was used to knock down TFDP3 expression in L-02 cell line and HepG2 cell line. We used siRNA2 and siRNA3 (both were effective TFDP3-siRNA constructed in our laboratory) to create the TFDP3 knockdown model. Western Blot result showed TFDP3-siRNA could knock-down the expression of TFDP3 in L-02 and HepG2 cell lines (Fig 4A). The statistical analysis of the relative gray value (TFDP3 / GAPDH) showed that the expression of TFDP3 in L-02 and HepG2 cell line was significantly down-regulated by the two siRNA sequences (Figure D in S1 Fig, n = 5). After establishing the TFDP3 knockdown model of the hepatocyte lines, the new cell cycles were detected by flow cytometry comparing with the untreated cells (Fig 4B). The results were analyzed and showed a significant decrease in G1 phase and increase in S phase in the TFDP3-knockdown groups (Figure E in S1 Fig, n = 5). It was demonstrated that knockdown of TFDP3 in L-02 and HepG2 cell line could induce the S phase blockage. Therefore, TFDP3 is involved in the process of G1 phase entry into S phase.

## Discussion

TFDP3, also known as HCA661, was a cancer-testis antigen (CTA), which originally identified by Dr. Chen in 2002 from human primary liver cancer tissue through SEREX technology[20]. Since van der et al. cloned the first cancer-testis antigen from tumor antigen-specific T cells and DNA clones in patients with melanoma, there were about 96 cancer-testis antigens have been found till now, which coded by 15 gene families and 31 genes. According to whether the antigens located on the X chromosome or not, the antigens could be divided into two categories, namely X-CTA, which located on the X chromosome; and Non X-CTA, which is not located on the X chromosome. Another characteristic of cancer-testis antigens is that their expression profile consists of normal testicular tissue and multiple types of malignancies. TFDP3 is located on the X chromosome, as the same as other cancer-testis antigens, only expressed in melanoma, hepatic carcinoma, breast cancer and T lymphocytic leukemia and other malignant tumor cells and normal testicular tissue[21]. The semi-quantitative PCR test to a variety of tissue proved this conclusion. In this study, the expression of TFDP3 in normal liver tissue was negative, and the expression of TFDP3 in hepatocellular carcinoma and testis was positive. Surprisingly, we found that TFDP3 was expressed in L-02 cell line, which is an immortalized human liver cell line. This phenomenon may be due to the normal liver tissue division activity is very weak, and immortalized human hepatocyte division activity is strong.

Cell cycle refers to the process from the end of a mitosis to the termination of next mitosis, which contains two stages, interphase and mitosis. Cell cycle plays an important role in the pathogenesis of many diseases and diseases. Interphase could be divided into three phases, namely G1phase, S phase and G2 phase. In G1 phase, large amounts of RNA and proteins would be synthesized in preparation for DNA synthesis[22]. S phase is the DNA synthesis period. In G2 phase, the protein synthesis would be completed to prepare into the mitosis. M phase could be divided into prophase, metaphase, anaphase and telophase. During this process, the agglutination of the chromosome is completed, the centrosomes move to the poles of nucleus, the dissolution of the nucleolus, the disappearance of the nucleolus (prophase), the spindle formation and chromosome arranged in the middle (metaphase), sister chromatid separated and moved toward the poles (anaphase), new nucleuses formation and cytoplasmic splitting (telophase). Cell cycle process is controlled by a variety of molecules with different mechanisms. The regular cell cycle plays an important role in regulating cell growth and proliferation. Blocking cell cycle has become a new clinical method for treatment of diseases or tumors[23].

Previous studies on TFDP family in cell cycle regulation focused more on the mechanism of the complex of TFDP1 and E2F family regulated cell cycle progression, and rarely mentioned the function of TFDP3 in cell cycle. Most of the studies on TFDP3 are focused on drug resistance and apoptosis. At present, some cancer-testis antigens are already used as tumor markers in tumor diagnosis, metastasis monitoring and tumor prevention. There are immune therapies based on cancer-testis antigens.

In this study, we discussed the function of TFDP3 in cell cycle regulation. We used TdR method to synchronize L-02 and HepG2 cell lines to G1, S, G2/M phases respectively. After that, we detect the expression of TFDP3 in L-02 and HepG2 cell line in each phases. The results showed that TFDP3 increased in S phase in both the cell lines. This may be due to the fact that activated E2F is no longer needed after the cells progress into S phase. In addition to the phosphorylation pathway, the inclined expression of TFDP3 in the S phase can compete with TFDP1 to bind to the activated E2F family members and inactivated them, thereby promoting cell progression into the next phase. This phenomenon is present in both immortalized hepatocytes and hepatoma cells.

Since the E2F or TFDP3 molecules alone cannot bind directly to DNA, TFDP3 must be combined with E2F family members to be in working. On the other hand, E2F molecules could hardly form to homologous complex. The general E2F complex is a heterodimeric complex formed by the binding of an E2F molecule and a DP molecule, then binding to the specific DNA sequence in the promoter region of the downstream target gene to initiate transcription and expression of the target gene, and eventually regulating cell proliferation and apoptosis and other activities[3]. TFDP3 molecules, like the E2F family and other members of the TFDP family, is predicted that closely related to cell cycle phase transition. E2F1 has been reported as an important regulatory factor in pRb pathway[24], which plays an important role in G1 phase to S phase in the cell cycle[25]. In addition to the pRb pathway, the transcriptional regulatory function of E2F1 is controlled by the p53-dependent negative feedback loop and the c-myc-regulated mir-RNA signal[26]. TFDP3 has a high sequence homology with TFDP1, which can inhibit E2F1 function by competing with TFDP1 to bind to E2F1 and affect the process of cell cycle. TFDP3 may also indirectly affect pRb and p53 pathway to be involved in the regulation of cell cycle. It still needed further experiments to confirm.

In the previous study, our research group had found that TFDP3 is related to the apoptosis and drug resistance of tumor cells, and its expression level may be related to the invasion ability of tumor cell lines. Therefore, we designed some specific knockdown TFDP3-siRNAs. The knockdown effect of TFDP3 was detected by Western blot. The TFDP3 knockdown model was successfully established in L-02 and HepG2 cell lines. After that, the percentage of cells in different phases in L-02 and HepG2 cell lines was measured by flow cytometry. The results showed that the percentage of G1 phase cells in L-02 and HepG2 cells in TFDP3 knockdown group was significantly lower than that in control group, indicating that TFDP3 affected cell progress into S phase during cell cycle. Since G1 phase is the only time that can be stimulated by the environment in the eukaryotic cell cycle, the restriction point at this period is the crux in the transition from G1 phase to S phase, which determines whether the cells continue to proliferate or stagnation or apoptosis. In this study, we found that the proportion of G1 phase cells in the TFDP3-knockdown group decreased and the proportion of S phase cells increased, however the G2/M phase cell changes were not significantly, which indicating that the knockdown of TFDP3 can induce S phase arrest. In addition, Qiao et al. demonstrated that TFDP3 overexpression could arrest cells in G1 phase, and the results of this study could further demonstrate that TFDP3 plays an important role in the cell cycle progression.

Targeting therapy for hepatic carcinoma is the necessary treatment for the prevention of recurrence. But it is still difficult to find specific cancer stem cell markers, as well as specific antibody treatments for them. This study demonstrates that TFDP3-knockdown siRNAs can block the cell cycle progression of L-02 and HepG2 cell lines, suggesting that TFDP3-siRNA is potentially useful in the treatment of malignant tumors such as hepatocellular carcinoma. Also, it is likely to be associated with chemotherapeutic drugs to become an important component in malignant tumor comprehensive treatment.

At present, cancer-testis antigens have been used as tumor markers for tumor diagnosis, metastasis monitoring and tumor prevention and treatment. The CTA-based tumor immunotherapy pathways have also been used in clinical trials. This study has already elucidated the important regulatory role of TFDP3 in the cell cycle progression, but the specific upstream and downstream signaling pathways in the process and the mechanism, as well as the specific structure and mechanism of inhibition of TFDP3 to E2F1 are still needed further study to be defined.

## Conclusion

This study confirmed that TFDP3 molecule is a cancer-testis antigen, and TFDP3 expresses in addition malignant tumor cell lines(such as HepG2), but also in the immortalized hepatocellular cell line(L-02). Its expression may related to the malignant situation of different cell lines and tissues, since it not expressed in normal liver tissue but expressed in cancer and testis tissues(Fig 1), and the expression level in hepatocyte immortality cell line(L-02) is lower than in hepatocellular carcinoma cell line(HepG2). The location of TFDP3 in L-02 cells and HepG2 cells was detected by immunofluorescence staining, and the expression level of TFDP3 expression was detected by Western blot. It can be seen that in L-02 and HepG2 cell lines, the expression level and position of TFDP3 is different (Fig 2).

We observed TFDP3 expression is characteristic in different phases in cell cycle and explored the mechanism. In this study, we used TdR method to synchronize the cell cycle, thus clarified the expression level and position of TFDP3 in L-02 cell line and HepG2 cell line at different phases(Fig 3). It showed that the TFDP3 molecule could bind to E2F1 in the process of cell cycle, which affected the process of cell cycle. It laid a foundation to further studying the mechanism of E2F/TFDP3 complex.

This study confirmed that TFDP3 is expected to be a potential target for the treatment of hepatic carcinoma. The use of TFDP3-siRNAs to knockdown the expression of TFDP3 in hepatocyte lines can influence the phase transition and process of cell cycle. To be specifically, it showed that the proportion of G1 phase was decreased and the proportion of S phase increased in L-02 and HepG2 cell lines. This result suggests that after TFDP3 was knocked down, the cells seemed to be blocked in the S phase, which is likely to increase the rate of apoptosis. Therefore, these TFDP3-siRNAs can affect the development of tumor cells by interfering cell cycle, which also provides the basis for the study of TFDP3-siRNAs in the treatment of TFDP3-positive tumors to extend the survival time of patients and improve the quality of life of patients.

## Supporting information

S1 Fig The statistical analysis of some figures in the paper. (A) The analysis of TFDP3 expression in L-02 and HepG2 cell lines. The relative gray scale values (TFDP3 / GAPDH) of L-02 and HepG2 were compared with student’s t test (n = 5). The gray value of Western Blot stripe measured by Image-Pro Plus software. The expression level of TFDP3 was higher in HepG2 cell line than in L-02 cell line (p = 0.036 <0.05). (B) The statistical analysis of the synchronization effect of L-02 and HepG2 cell lines to G1, S, G2 and M phase (n = 5). (C) The analysis of TFDP3 expression in L-02 and HepG2 cell lines in different phase in cell cycle. ** indicates a significant difference when compared to the negative control group at p <0.01. The expression level in S phase was higher than the other phases (p_L-02_ = 0.003<0.01; p_HepG2_ = 0.007<0.01; n = 5) (D) The analysis of TFDP3 knockdown effect in L-02 and HepG2 cell lines. The statistical analysis of the relative gray value (TFDP3 / GAPDH) showed that the expression of TFDP3 in L-02 and HepG2 cell line was significantly down-regulated by the two siRNA sequences (n = 5). * indicates a significant difference when compared to the negative control group at p <0.05; ** indicates a significant difference when compared to the negative control group at p <0.01. The expression of TFDP3 was significantly lower than that of the control group after transfection of TFDP3-siRNA2 and TFDP3-siRNA3 in L-02 and HepG2 cell lines, indicating that TFDP3 knockdown model was established successfully. (E) The comparison of cell proportion of every phase in cell cycle before and after TFDP3 knockdown was analyzed (n = 5). It is significantly that the cell proportion in G1 phase decrease, and the proportion in S phase increase (p<0.05).
(TIF)

S1 File The raw data of some figures and tables in the paper. It is the catalog of files in the zip file below:
Folder A: The uncropped Western blot images of Fig 2B, Fig 3B and Fig 4A.
Figure A: The uncropped Western blot image of Fig 2B (GAPDH).
Figure B: The uncropped Western blot image of Fig 2B (TFDP3).
Figure C: The uncropped Western blot image of Fig 2B with tags.
Figure D: The uncropped Western blot image of Fig 3B (HepG2).
Figure E: The uncropped Western blot image of Fig 3B (L-02 GAPDH).
Figure F: The uncropped Western blot image of Fig 3B (L-02 TFDP3).
Figure G: The uncropped Western blot image of Fig 3B with tags.
Figure H: The uncropped Western blot image of Fig 4A.
Figure I: The uncropped Western blot image of Fig 4A with tags.
Folder B: The raw data of Fig 2A and S1 Fig.
Table A: The raw data of Fig 2A.
Table B: The raw data of S1 Fig.
(RAR)
