# Supplementary figures and images for: Effects of cancer-testis antigen, TFDP3, on cell cycle regulation and its mechanism in L-02 and HepG2 cell lines in vitro
Source: PLoS One. 2017 Aug 10;12(8):e0182781. doi: 10.1371/journal.pone.0182781 (PMC5552311; doi:10.1371/journal.pone.0182781)

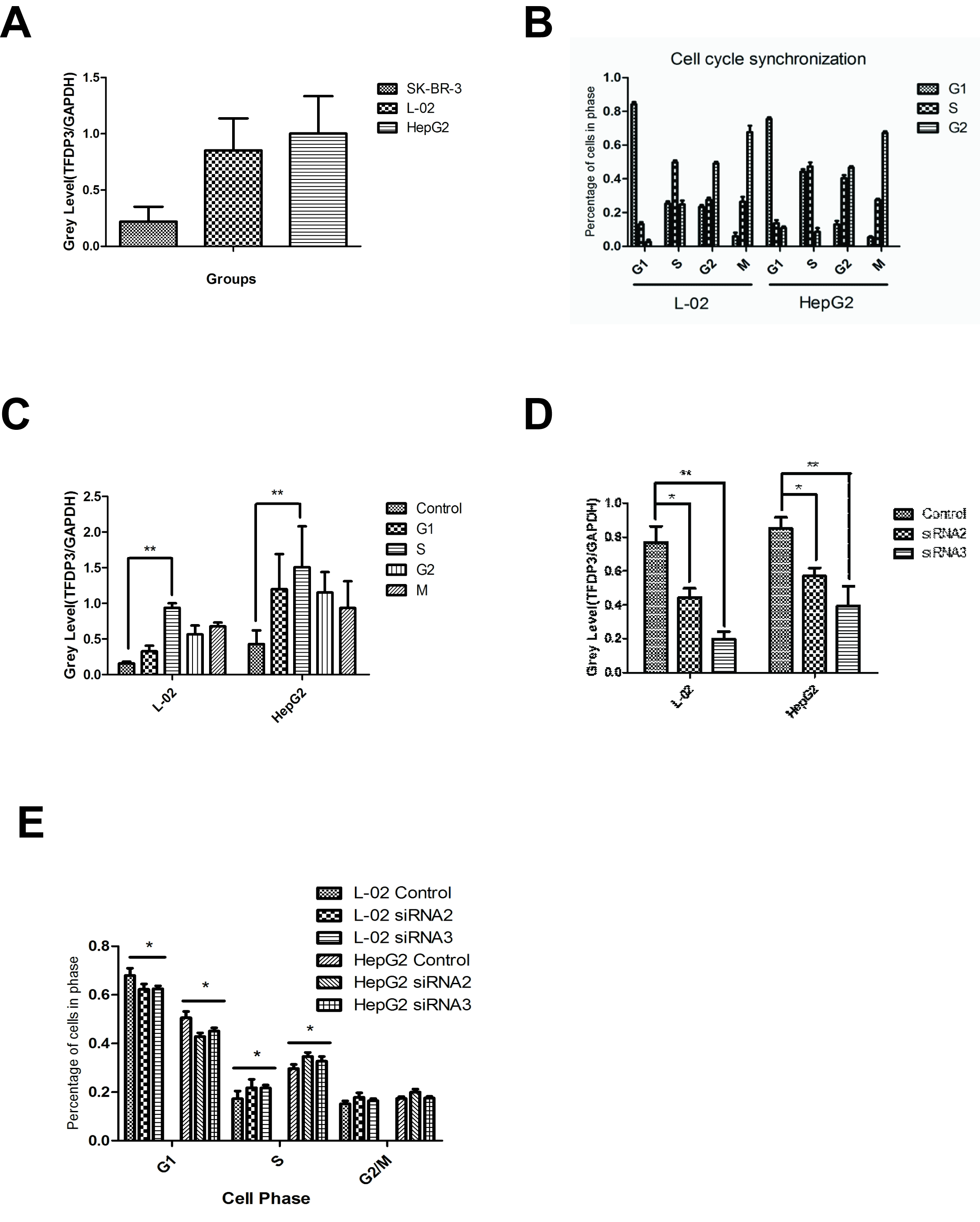

Supplement: S1 Fig — (A) The analysis of TFDP3 expression in L-02 and HepG2 cell lines. The relative gray scale values (TFDP3 / GAPDH) of L-02 and HepG2 were compared with student’s t test (n = 5). The gray value of Western Blot stripe measured by Image-Pro Plus software. The expression level of TFDP3 was higher in HepG2 cell line than in L-02 cell line (p = 0.036 <0.05). (B) The statistical analysis of the synchronization effect of L-02 and HepG2 cell lines to G1, S, G2 and M phase (n = 5). (C) The analysis of TFDP3 expression in L-02 and HepG2 cell lines in different phase in cell cycle. ** indicates a significant difference when compared to the negative control group at p <0.01. The expression level in S phase was higher than the other phases (pL-02 = 0.003<0.01; pHepG2 = 0.007<0.01; n = 5) (D) The analysis of TFDP3 knockdown effect in L-02 and HepG2 cell lines. The statistical analysis of the relative gray value (TFDP3 / GAPDH) showed that the expression of TFDP3 in L-02 and HepG2 cell line was significantly down-regulated by the two siRNA sequences (n = 5). * indicates a significant difference when compared to the negative control group at p <0.05; ** indicates a significant difference when compared to the negative control group at p <0.01. The expression of TFDP3 was significantly lower than that of the control group after transfection of TFDP3-siRNA2 and TFDP3-siRNA3 in L-02 and HepG2 cell lines, indicating that TFDP3 knockdown model was established successfully. (E) The comparison of cell proportion of every phase in cell cycle before and after TFDP3 knockdown was analyzed (n = 5). It is significantly that the cell proportion in G1 phase decrease, and the proportion in S phase increase (p<0.05). (TIF) [file pone.0182781.s001.tif]
